# Olomoucine II, but Not Purvalanol A, Is Transported by Breast Cancer Resistance Protein (ABCG2) and P-Glycoprotein (ABCB1)

**DOI:** 10.1371/journal.pone.0075520

**Published:** 2013-10-08

**Authors:** Jakub Hofman, Radim Kučera, Daniela Cihalova, Jiri Klimes, Martina Ceckova, Frantisek Staud

**Affiliations:** 1 Department of Pharmacology and Toxicology, Charles University in Prague, Faculty of Pharmacy, Hradec Kralove, Czech Republic; 2 Department of Pharmaceutical Chemistry and Drug Analysis, Charles University in Prague, Faculty of Pharmacy, Hradec Kralove, Czech Republic; University of Cambridge, United Kingdom

## Abstract

Purine cyclin-dependent kinase inhibitors have been recognized as promising candidates for the treatment of various cancers; nevertheless, data regarding interaction of these substances with drug efflux transporters is still lacking. Recently, we have demonstrated inhibition of breast cancer resistance protein (ABCG2) by olomoucine II and purvalanol A and shown that these compounds are able to synergistically potentiate the antiproliferative effect of mitoxantrone, an ABCG2 substrate. In this follow up study, we investigated whether olomoucine II and purvalanol A are transported by ABCG2 and ABCB1 (P-glycoprotein). Using monolayers of MDCKII cells stably expressing human ABCB1 or ABCG2, we demonstrated that olomoucine II, but not purvalanol A, is a dual substrate of both ABCG2 and ABCB1. We, therefore, assume that pharmacokinetics of olomoucine II will be affected by both ABCB1 and ABCG2 transport proteins, which might potentially result in limited accumulation of the compound in tumor tissues or lead to drug-drug interactions. Pharmacokinetic behavior of purvalanol A, on the other hand, does not seem to be affected by either ABCG2 or ABCB1, theoretically favoring this drug in the potential treatment of efflux transporter-based multidrug resistant tumors. In addition, we observed intensive sulfatation of olomoucine II in MDCKII cell lines with subsequent active efflux of the metabolite out of the cells. Therefore, care should be taken when performing pharmacokinetic studies in MDCKII cells, especially if radiolabeled substrates are used; the generated sulfated conjugate may largely contaminate pharmacokinetic analysis and result in misleading interpretation. With regard to chemical structures of olomoucine II and purvalanol A, our data emphasize that even drugs with remarkable structure similarity may show different pharmacokinetic behavior such as interactions with ABC transporters or biotransformation enzymes.

## Introduction

Olomoucine II and purvalanol A are potent cyclin-dependent kinase inhibitors (CDKi) that belong to the group of 2,6,9-trisubstituted purine derivatives [1], [2]. These compounds effectively stop cellular proliferation, block transcription of essential genes and induce apoptosis [3]–[5]. For their favorable pharmacodynamic properties, purine CDKi have become modern alternatives in cancer therapy [6], [7]. Roscovitine (seliciclib, CYC202), a structural analogue of olomoucine II and purvalanol A, has reached phase II trials for treating various cancers [8], [9]. Although olomoucine II and purvalanol A are commonly considered selective for cyclin-dependent kinases, several studies have reported their subordinate intracellular targets from the superfamily of protein kinases, which are inhibited by these compounds in the range of micromolar concentrations [3], [10]–[13]. However, possible interactions with other biological structures, such as drug transporters, have not been properly investigated to date.

ATP-binding cassette transporters (ABC transporters) are membrane proteins that pump many structurally unrelated molecules, including drugs and toxins, out of cells. The most widely studied members of this family, P-glycoprotein (ABCB1) and breast cancer resistance protein (ABCG2), are abundantly expressed in absorptive and eliminatory organs (e.g. small intestine, liver, kidney) as well as in several blood-tissue barriers (e.g. blood-brain barrier, placenta, blood-testis barrier) playing crucial role in drug disposition [14], [15]. In addition, by diminishing intracellular concentrations of chemotherapeutics in cancer cells, ABCB1 and ABCG2 transporters are frequently associated with the multidrug resistance phenomenon [16], [17]. Modulation of these transporters is, therefore, of great clinical interest; ABC transporter inhibitors have been investigated for their ability to restore the sensitivity of tumor cells to chemotherapy or to increase oral bioavailability and tissue penetration of ABC transporter substrates [18]–[20]. Moreover, investigating interactions of novel drug entities with transport proteins is an important issue in drug discovery and development [21].

Recently, we have demonstrated inhibition of ABCG2 by olomoucine II, purvalanol A, bohemine and roscovitine at *in vitro* and *in situ* levels [22]. Olomoucine II and purvalanol A showed comparable or even higher potency than fumitremorgin C, a model specific ABCG2 inhibitor. Moreover, using combination method of Chou-Talalay, we demonstrated that these compounds can synergistically potentiate the antiproliferative effect of mitoxantrone, an ABCG2 substrate, in ABCG2-expressing cell lines [22]. In the present paper, we employed transport assays in MDCKII cells stably expressing ABCG2 or ABCB1 to investigate whether transcellular passage of olomoucine II and purvalanol A is affected by these transporters.

## Materials and Methods

### Reagents and Chemicals

Olomoucine II and purvalanol A were purchased from Sigma-Aldrich (St. Louis, MO, USA). Specific ABCG2 inhibitor, fumitremorgin C, was supplied by Alexis Corporation (Lausanne, Switzerland). Specific ABCB1 inhibitor, LY335979, was obtained from Toronto Research Chemicals (North York, ON, Canada). Cell culture reagents were obtained from Sigma Aldrich (St. Louis, MO, USA) and from Gibco BRL Life Technologies (Rockville, MD, USA). Fluorescein isothiocyanate labeled dextran was from Sigma-Aldrich (St. Louis, MO, USA). All other compounds and agents were of analytical grade.

### Cell Cultures

ABCG2- and ABCB1-transduced MDCKII sublines (MDCKII-ABCG2 and MDCKII-ABCB1), which stably express ABCG2 and ABCB1 protein, respectively, were purchased from dr. Alfred Schinkeĺs lab (The Netherlands Cancer Institute, Amsterdam, The Netherlands). These transduced sublines as well as the parental MDCKII cell line (MDCKII-par) were routinely cultured in complete Dulbecco’s modified Eagle’s medium with 10% fetal bovine serum. 100 U/ml penicillin and 100 µg/ml streptomycin were used while growing the cells on the membrane inserts. All cells were routinely cultivated in antibiotic-free medium and periodically tested for mycoplasma contamination. Stable expression of ABCB1 and ABCG2 was verified by qRT-PCR method and by daunorubicin and mitoxantrone efflux activity, respectively. Cells from passages 15 to 25 were used in all *in vitro* studies. Dimethyl sulfoxide was applied as a CDKi solvent in concentrations not exceeding 0.1%.

### Cellular Monolayer Transport Assay

Transport assays were performed on microporous polycarbonate membrane inserts (3 µm pore size, 24 mm diameter; Transwell 3414, Costar, Cambridge, MA, USA) as described previously [22]. MDCKII-ABCG2, MDCKII-ABCB1 or MDCKII-par cells were seeded at a density of 1×10^6^ per insert 72 h before experiment. The medium was replaced after 24 and 48 h of cultivation. One hour before the start of the experiment, the cells were washed with prewarmed 1×phospate buffered saline on both the apical and basal sides and Opti-MEM with or without fumitremorgin C or LY335979 was added into both compartments. At time 0, the experiment was started by replacing the medium with fresh Opti-MEM with or without CDKi and fumitremorgin C or LY335979 in the appropriate chamber. Samples were taken every 2 h from the opposite chambers for the duration of the experiment (6 h). Concentration of CDKi was determined via HPLC/MS analysis. Immediately after the experiment, cellular monolayer integrity was examined using fluorescein isothiocyanate labeled dextran (MW = 40 kDa). Dextran leakage was accepted up to 1% per hour.

### HPLC/MS Analysis

HPLC/MS analysis using LC 20A Prominence chromatographic system (Shimadzu, Kyoto, Japan) coupled with LCQ Max advantage mass spectrometer (Thermo Finnigan, San Jose, CA, USA) was used for the quantification of olomoucine II and purvalanol A. The separation was performed on a Hypersil GOLD C18 column (100 × 4.6 mm, particle size 3 µm) protected with an OPTI-GUARD 1 mm guard column C18. The mobile phase flow rate was 0.35 ml/min and the column temperature was maintained at 40°C. The data were processed using Xcalibur 2.0 software (Thermo Finnigan, San Jose, CA, USA).

Optimal separation of olomoucine II was achieved in mobile phase containing the mixture of methanol and 0.0125% formic acid (62∶38, v/v). Bohemine was added to samples as the internal standard (IS). Retention times were 5.5 and 6.5 min for IS and olomoucine II, respectively. The detector was set as follows: spray voltage of 4.5 kV, capillary temperature of 320°C, sheet and auxiliary gas flows of 30 and 12 arbitrary units, respectively. The chromatograms were recorded in SRM mode using precursor ion at [M+H]^+^ (*m/z:* 371 olomoucine II and 341 IS) and the product ions 265 (olomoucine II) and 250 (IS) were used for quantification after collision dissociation. The collision energies were 38% and 40% for olomoucine II and IS, respectively. The linearity of the method was evaluated in the range of 5–500 µM (r^2^ = 0.9961); the method precision and accuracy were evaluated at 500, 100, 10 and 5 nM. The sample stability was evaluated within 94 h.

A mixture of methanol and 0.01% acetic acid (75∶25 v/v) was used for the separation of purvalanol A. Roscovitine was utilized as the IS. Retention times were 5.7 and 9.2 min for IS and purvalanol A, respectively. The detector was set as follows: spray voltage of 5.5 kV, capillary temperature of 340°C, sheet and auxiliary gas flows of 28 and 13 arbitrary units, respectively. The chromatograms were recorded in SRM mode using precursor ion at [M+H]^+^ (*m/z:* 389 purvalanol A and 355 IS) and the product ions 303 (purvalanol A) and 312 (IS) were used for the quantification after collision dissociation. The collision energies were 38% and 40% for purvalanol A and IS, respectively. The linearity of the method was evaluated in the range of 15–384 µM (r^2^ = 0.9925); the method precision and accuracy were evaluated at 384, 100 and 15 nM. The sample stability was evaluated within 94 hours.

### Statistical Analysis

Student’s *t* test was used to assess statistical significance for *in vitro* monolayer transport assays. Differences of p<0.05 were considered statistically significant.

## Results

### Effect of ABCG2 and ABCB1 on the Transepithelial Transport of Olomoucine II *In Vitro*

Transport of olomoucine II by ABCG2 and ABCB1 was tested *in vitro* using transport assays across the polarized monolayers of MDCKII-ABCG2 and MDCKII-ABCB1 cells, respectively. In this method, the transport across the monolayer is greatly accelerated in the basolateral to apical direction, when the compound is a substrate of examined transporter. Based on our previous studies [22], three olomoucine II concentrations (100 nM, 1 µM and 10 µM) were tested and transport ratios (*r*; olomoucine II transport in basolateral to apical direction divided by transport in apical to basolateral direction) 2 h after olomoucine II addition were calculated. In contrast to purvalanol A, the interval for data evaluation was shortened due to the generation of sulfated metabolite of olomoucine II (see below).

**Figure 1:**
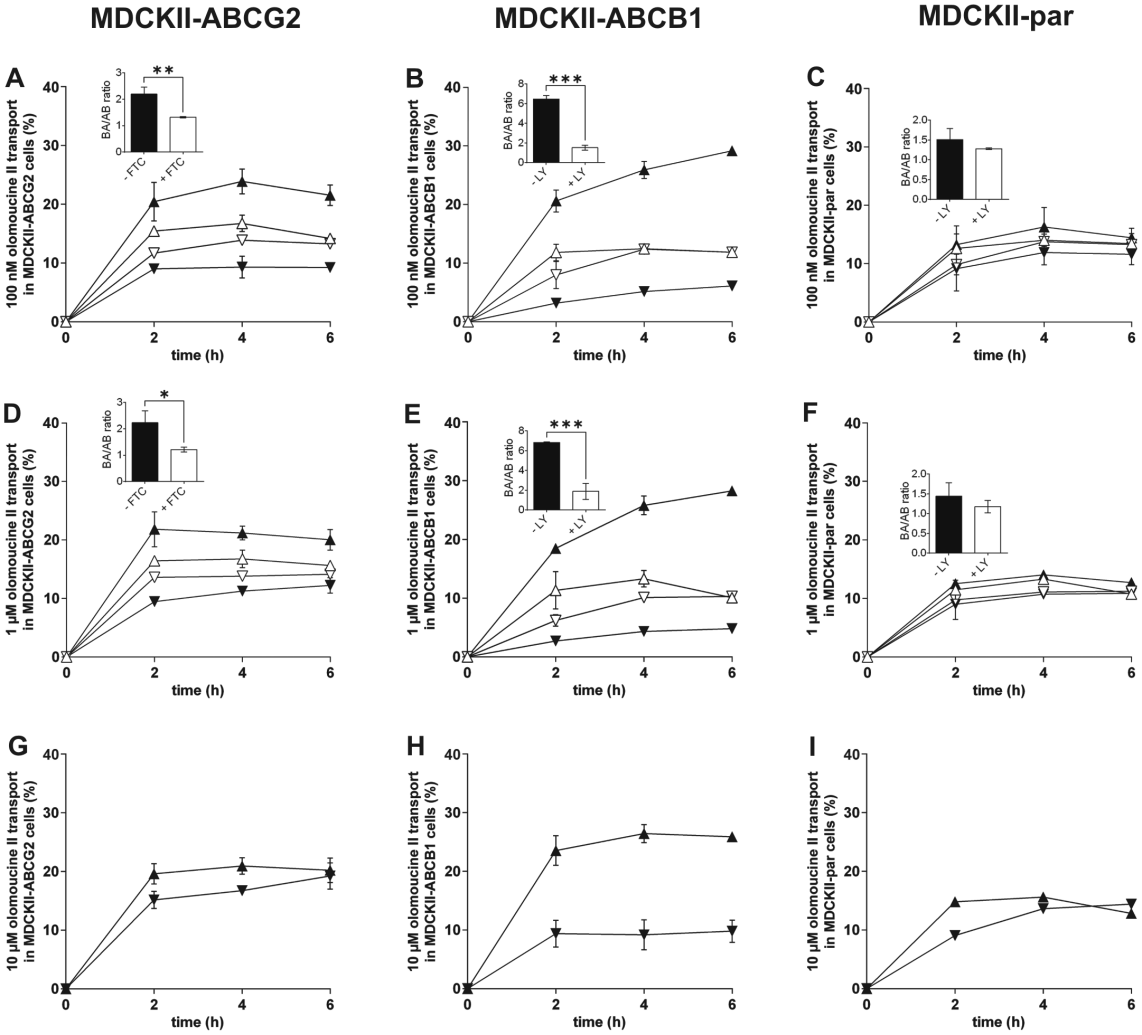
Transport of olomoucine II at concentrations of 100(A, B, C), 1 µM (D, E, F) and 10 µM (G, H, I) across monolayers of MDCKII-ABCG2 (A, D, G), MDCKII-ABCB1 (B, E, H) and MDCKII-par (C, F, I) cells. 5 µM fumitremorgin C (FTC) was used as a specific ABCG2 inhibitor in MDCKII-ABCG2 cells. 1 µM LY335979 (LY) was employed as a specific ABCB1 and endogenous canine Abcb1 inhibitor in MDCKII-ABCB1 and MDCKII-par cells, respectively. Ratios of olomoucine II transport across cell monolayers (olomoucine II transport in basolateral to apical direction divided by transport in apical to basolateral direction) with or without inhibitor were calculated two hours after olomoucine II addition and statistically compared (see insets). Due to the generation of sulfated conjugate of olomoucine II, transport ratios were determined at 2 h interval to reduce the misrepresenting effect of the metabolite. In basolateral to apical transport direction, olomoucine II was added into the basolateral compartment and its concentrations were determined in the apical compartment. In the opposite transport direction, olomoucine II was applied into the apical compartment and its concentrations were analyzed in the basolateral compartment. ▴, basolateral to apical transport without inhibitor; ▾, apical to basolateral transport without inhibitor; ▵, basolateral to apical transport with inhibitor; ▿, apical to basolateral transport with inhibitor. Data are expressed as means ± SD of three independent experiments. *p<0.05; **p<0.01; ***p<0.001.

Using olomoucine II in concentrations of 100 nM or 1 µM, similar *r* values equal to 2.27 and 2.31 were observed in MDCKII-ABCG2 cells, respectively. Fumitremorgin C, a specific ABCG2 inhibitor [23], significantly lowered asymmetry in olomoucine II transport to *r* values of 1.32 and 1.21, respectively, thereby confirming the involvement of ABCG2 in the transport of olomoucine II (Fig. 1A, 1D). When 10 µM olomoucine II was used, *r* decreased to 1.29, indicating transporter saturation (Fig. 1G).

In MDCKII-ABCB1 cells, asymmetry in olomoucine II transport was approximately 3-fold higher (*r* = 6.45 and 6.83 for 100 nM and 1 µM, respectively) in comparison with ABCG2 transduced cells. LY335979, a specific ABCB1 inhibitor [24], [25], significantly reduced *r* to values similar to those observed in the case of parent MDCKII cells (*r* = 1.48 and 1.83 for 100 nM and 1 µM, respectively) (Fig. 1B, 1E). At 10 µM concentration, *r* decreased to 2.51 indicating partial saturation of ABCB1 transporter (Fig. 1H). These results clearly demonstrate that olomoucine II is a substrate of ABCB1 *in vitro*.

In MDCKII-par cells, slight transport asymmetry was found with *r* values of 1.45, 1.39 and 1.64 for 100 nM, 1 µM and 10 µM olomoucine II, respectively (Fig. 1C, 1F, 1I). Since MDCKII-par cells express significant amount of endogenous canine Abcb1 [26], [27], we investigated its possible role in this basal transport by adding LY335979 inhibitor. However, we did not observe statistically significant changes in *r* values for 100 nM and 1 µM olomoucine II, suggesting no or negligible participation of endogenous canine Abcb1 in olomoucine II transport (Fig. 1C, 1F).

### Generation of Sulfated Conjugate of Olomoucine II and its Pharmacokinetic Behavior in MDCKII Cells

**Figure 2:**
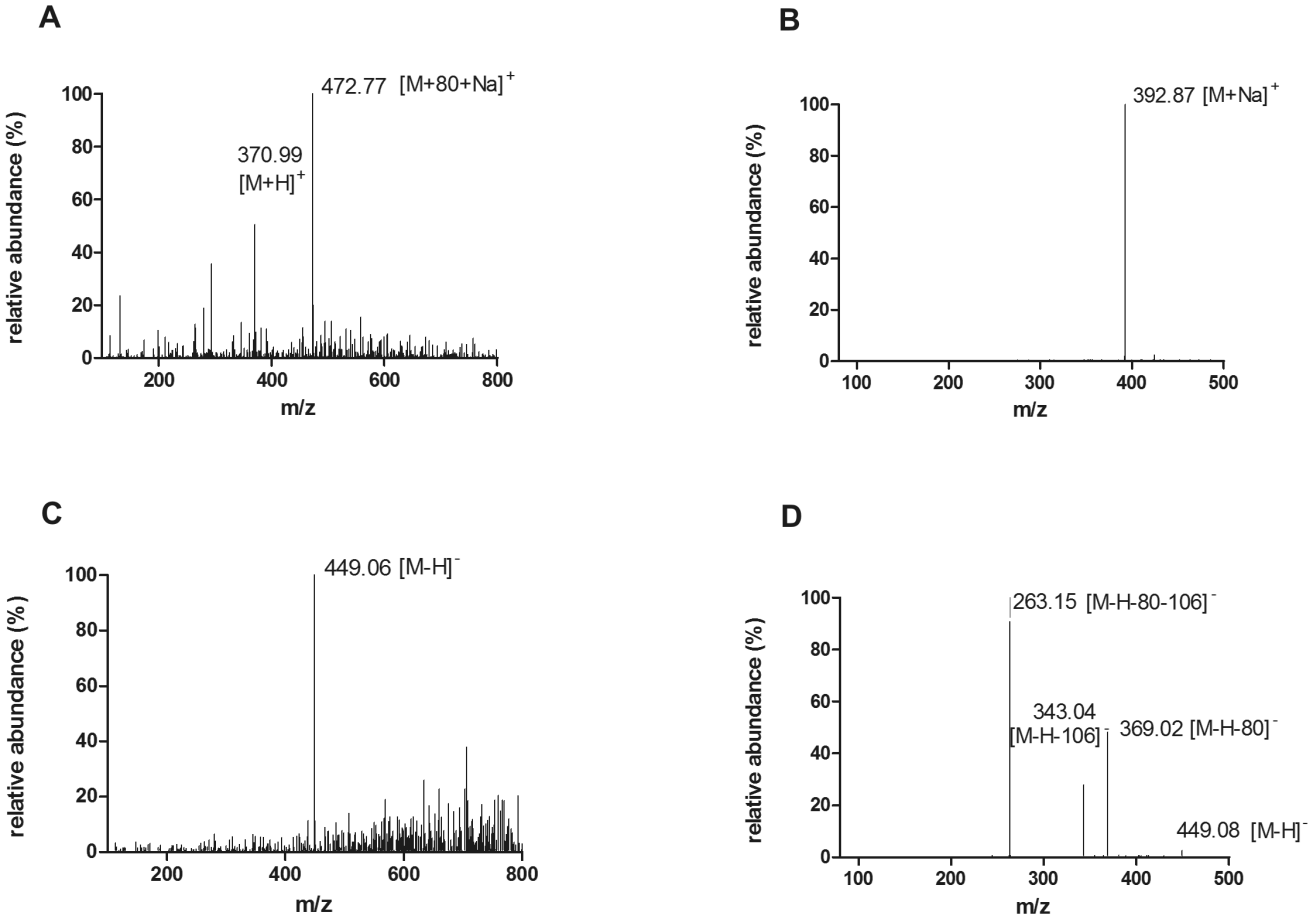
Mass spectra of an unknown peak eluted in the fifth minute of HPLC analysis of olomoucine II transport. (A) spectrum in positive mode, (B) MS^2^ in positive mode, (C) negative mode, (D) MS^2^ in negative mode. Based on the nominal mass shift (+80 Da) from parent compound and the collision spectra in negative as well as positive mode the compound was identified as a sulfated conjugate of olomoucine II.

**Figure 3:**
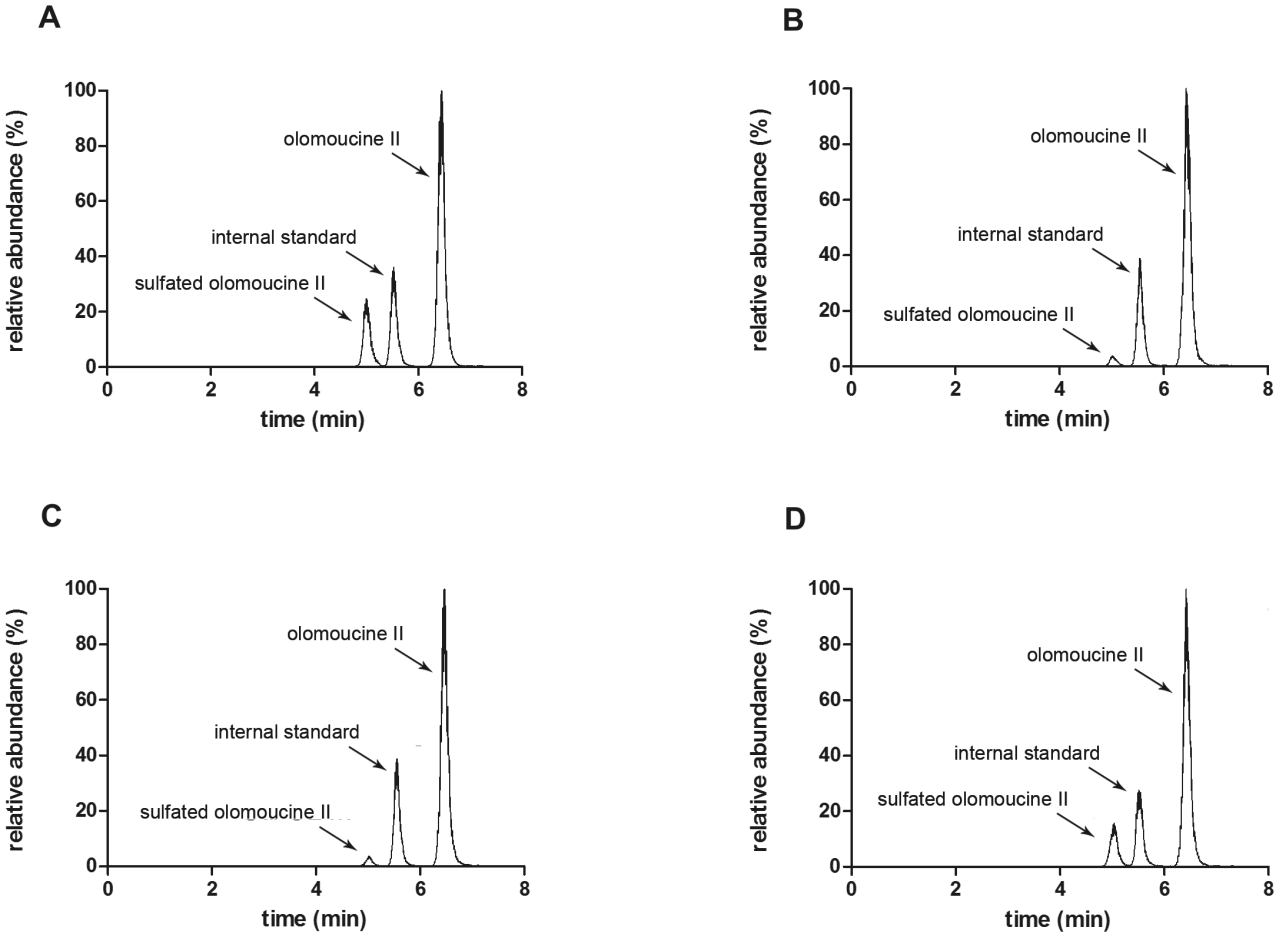
Chromatograms of samples from MDCKII-par cells six hours after olomoucine II addition. (A) olomoucine II was added into apical compartment while olomoucine II and its sulfated conjugate were analyzed in acceptor basolateral compartment, (B) olomoucine II was added into apical compartment while olomoucine II and its sulfated conjugate were analyzed in donor apical compartment, (C) olomoucine II was added into basolateral compartment while olomoucine II and its sulfated conjugate were analyzed in acceptor apical compartment, (D) olomoucine II was added into basolateral compartment while olomoucine II and its sulfated conjugate were analyzed in donor basolateral compartment. This analysis with end point samples was performed for all olomoucine II transport experiments.

In olomoucine II transport experiments, we recorded time-dependent generation of an unknown peak in the fifth minute of HPLC analysis in all three cell lines tested (MDCKII-ABCG2, MDCKII-ABCB1 and MDCKII-par). Based on the MS analysis [28], the compound was identified as a sulfated conjugate of olomoucine II (Fig. 2). Importantly, equilibrium of sulfate conjugate distribution into particular compartments did not significantly differ when the parent compound was added into the basal or apical compartment (Fig. 3). On the other hand, the amount of sulfated metabolite increased with time and caused remarkable distortion of the results of parent compound. Therefore, when analyzing the data of unconjugated olomoucine II, the time interval was shortened to 2 hours in order to reduce the misrepresenting effect of sulfated olomoucine II. Considering ionic nature of sulfated olomoucine II, it is obvious that this compound cannot escape from the cells via passive diffusion but must utilize special transport system(s). Relative quantification of olomoucine II sulfate allowed us to describe its pharmacokinetic behavior in all MDCKII cell sublines.

**Figure 4:**
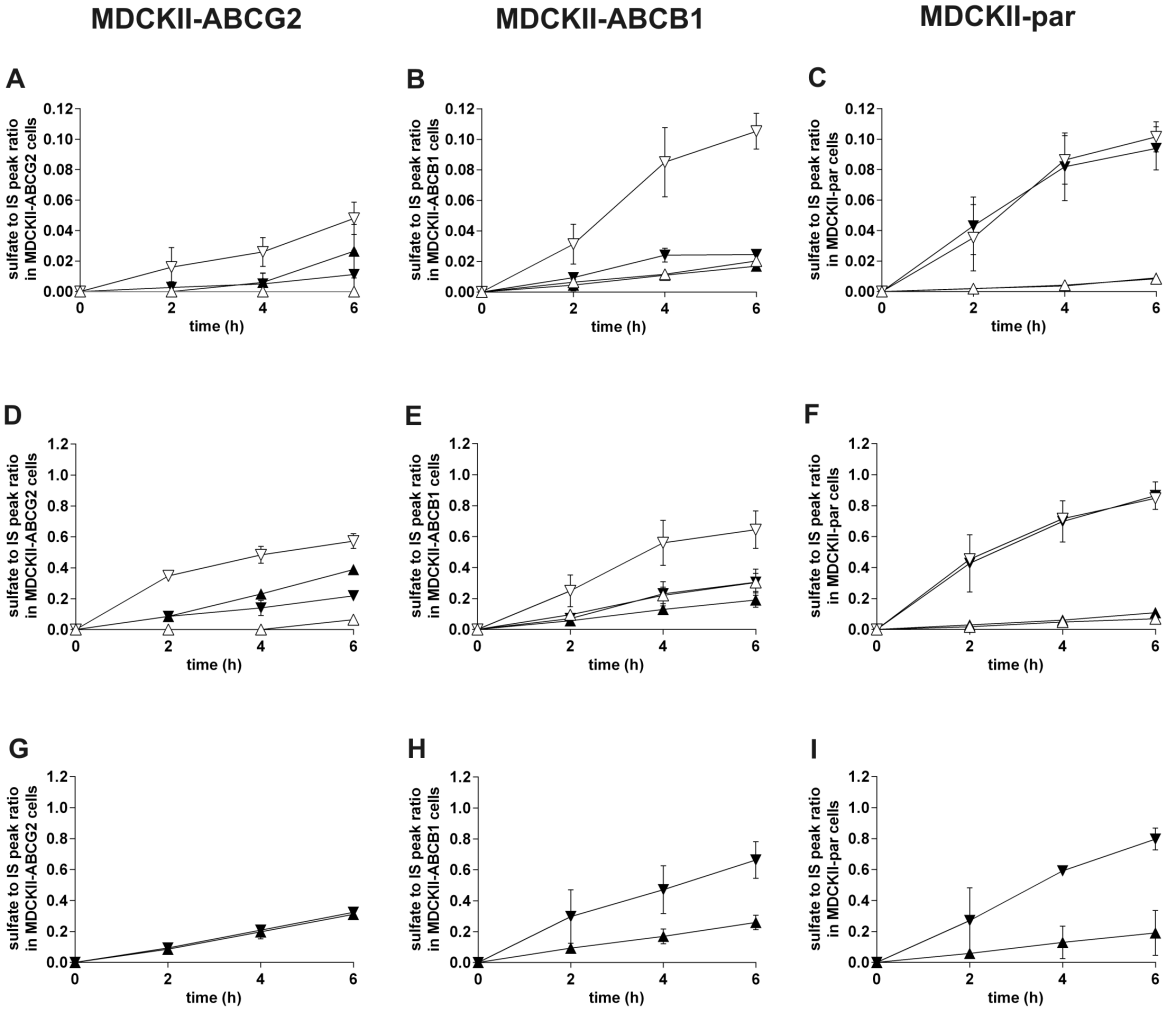
Time-dependent generation of sulfated conjugate of olomoucine II in MDCKII-ABCG2 (A, D, G), MDCKII-ABCB1 (B, E, H) and MDCKII-par (C, F, I) cells and its distribution into the apical and basolateral compartments. Relative quantification of sulfated olomoucine II was calculated as a ratio between peak area of sulfated olomoucine II and the peak area of internal standard (IS). 5 µM fumitremorgin C (FTC), a specific ABCG2 inhibitor, was used in MDCKII-ABCG2 cells for the assessment of possible involvement of ABCG2 in the transport of sulfated metabolite. 1 µM LY335979 (LY) was employed as a specific ABCB1 and endogenous canine Abcb1 inhibitor in MDCKII-ABCB1 and MDCKII-par cells, respectively. Data come from transport experiments with olomoucine II at concentrations of 100 nM (A, B, C), 1 µM (D, E, F) and 10 µM (G, H, I). In basolateral to apical transport direction, olomoucine II was added into the basolateral compartment and its sulfate conjugate was determined in the apical compartment. In the opposite transport direction, olomoucine II was applied into the apical compartment and its sulfated metabolite was analyzed in the basolateral compartment. ▴, transport into apical compartment without inhibitor; ▾, transport into basolateral compartment without inhibitor; ▵, transport into apical compartment with inhibitor; ▿, transport into basolateral compartment with inhibitor. Values are expressed as means ± SD of three independent experiments.

In MDCKII-ABCG2 cells at 100 nM olomoucine II concentration, the sulfate appearance in apical compartment was markedly higher than that in basolateral compartment. After addition of fumitremorgin C, efflux into apical compartment was fully blocked whereas transport into opposite compartment significantly increased (Fig. 4A). Similar outcome was recorded in the case of 1 µM olomoucine II (Fig. 4D). These data demonstrate the contribution of ABCG2 to the efflux of sulfated olomoucine II through the apical membrane of MDCKII-ABCG2 cells. At 10 µM concentration, we observed identical appearance of sulfated conjugate in both compartments (Fig. 4G), suggesting saturation of ABCG2.

In MDCKII-ABCB1 cells at 100 nM and 1 µM olomoucine II, distribution of sulfated olomoucine II into basolateral and apical compartments was almost identical. This equilibrium was notably changed after LY335979 co-administration which increased appearance of the conjugate in the basolateral, but not apical, compartment (Fig. 4B, 4E). This phenomenon can be explained by LY335979-induced inhibition of ABCB1 which results in higher intracellular concentrations of olomoucine II and, therefore, greater availability of the drug for sulfatation. Generated olomoucine II sulfate is eventually transferred into basolateral compartment, most likely by endogenous canine transporters reported in MDCKII cells [26]. Sulfate distribution ratio after LY335979 addition in MDCKII-ABCB1 cells was almost identical to that observed in MDCKII-par, supporting this hypothesis.

Transport of olomoucine II sulfate in MDCKII-par cells, expressing only endogenous canine transporters, was markedly forced into the basolateral compartment whereas only limited amount reached the apical one. Addition of LY335979 did not affect this asymmetry, excluding the role of canine Abcb1 in the process (Fig. 4C, 4F, 4I). We, therefore, speculate that a transport system located in the basolateral membrane is the key player affecting metabolite distribution in non-transduced cells.

### Effect of ABCG2 and ABCB1 on the Transepithelial Transport of Purvalanol A *In Vitro*

Possible involvement of ABCG2 and/or ABCB1 in the transcellular transport of purvalanol A was examined employing cellular monolayer transport assays with MDCKII-ABCG2 and MDCKII-ABCB1 cells, respectively. Based on our previous studies [22], two purvalanol A concentrations (1 µM and 10 µM) were tested and transport ratios (*r*; purvalanol A transport in basolateral to apical direction divided by transport in apical to basolateral direction) at the end of the experiment were calculated.

**Figure 5:**
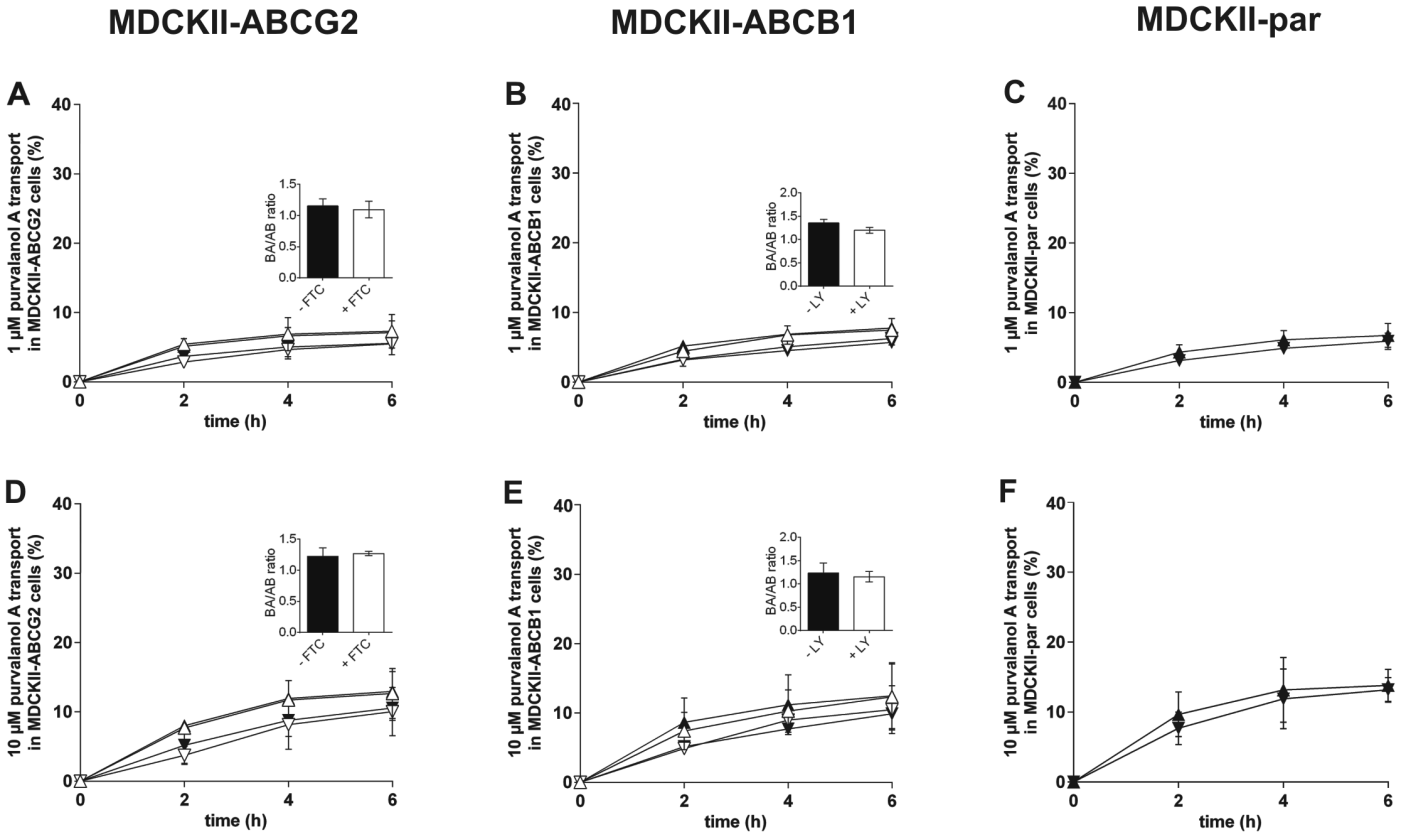
Transport of purvalanol A at concentrations of 1 µM (A, B, C) and 10 µM (D, E, F) across monolayers of MDCKII-ABCG2 (A, D), MDCKII-ABCB1 (B, E) and MDCKII-par (C, F) cells. 5 µM fumitremorgin C (FTC) was used as a specific ABCG2 inhibitor in MDCKII-ABCG2 cells. 1 µM LY335979 (LY) was employed as a specific ABCB1 inhibitor in MDCKII-ABCB1 cells. Ratios of purvalanol A transport across cell monolayers (purvalanol A transport in basolateral to apical direction divided by transport in apical to basolateral direction) with or without inhibitor were calculated and statistically compared (see insets). Transport ratios were determined 6 h after purvalanol A addition. In basolateral to apical transport direction, purvalanol A was added into the basolateral compartment and its concentrations were determined in the apical compartment. In the opposite transport direction, purvalanol A was applied into the apical compartment and its concentrations were analyzed in the basolateral compartment. ▴, basolateral to apical transport without inhibitor; ▾, apical to basolateral transport without inhibitor; ▵, basolateral to apical transport with inhibitor; ▿, apical to basolateral transport with inhibitor. Data are expressed as means ± SD of three independent experiments.

In contrast to olomoucine II, only negligible asymmetry in purvalanol A transport was observed in MDCKII-ABCG2 cells with *r* of 1.28 and 1.23 for 1 µM and 10 µM, respectively. No changes were recorded after concomitant addition of fumitremorgin C (*r* = 1.33 and 1.26 for 1 µM and 10 µM, respectively) (Fig. 5A, 5D). These results demonstrate that purvalanol A is not an ABCG2 substrate *in vitro*.

Similar results were obtained in MDCKII-ABCB1 cells; only negligible asymmetry in purvalanol A transport was observed, reaching *r* values of 1.36 and 1.26 for 1 µM and 10 µM, respectively. Addition of LY335979 did not affect *r* values (1.20 and 1.18 for 1 µM and 10 µM, respectively) (Fig. 5B, 5E). These patterns of transport clearly demonstrate that purvalanol A is not an ABCB1 substrate.

As expected, only negligible asymmetry in purvalanol A transport was observed in MDCKII-par cells. 1 µM and 10 µM purvalanol A concentrations yielded *r* values of 1.14 and 1.05, respectively (Fig. 5C, 5F).

Interestingly, no fragments corresponding to sulfated purvalanol A were recorded in MS analysis indicating that, in contrast to olomoucine II, purvalanol A is not subjected to sulfatation in MDCKII cells.

## Discussion

Purine CDKi have recently been recognized as promising candidates for the treatment of various cancers [6]. While pharmacodynamic properties of these compounds are relatively well understood, their pharmacokinetic behavior and interactions with other biological structures, such as transport proteins and biotransformation enzymes have not been properly investigated to date. Bachmaier and Miller were the first to observe interactions of purine CDKi with ABC transporters and demonstrated significant inhibition of ABCB1 by roscovitine in bovine brain microvessel endothelial cell monolayers [29]. More recently, An et al. revealed ABCG2 inhibition by purvalanol A, WHI-P180, roscovitine and bohemine employing *in vitro* hematoporphyrin transport across membrane vesicles from insect Sf9 cells transduced with ABCG2 [30]. In our previous work, we observed ABCG2 inhibition by olomoucine II and purvalanol A on *in vitro* as well as *in situ* level. Moreover, using combination method of Chou-Talalay, we demonstrated that these compounds can synergistically potentiate the cytostatic effect of mitoxantrone, an ABCG2 substrate, in ABCG2 expressing cell lines [22].

**Figure 6:**
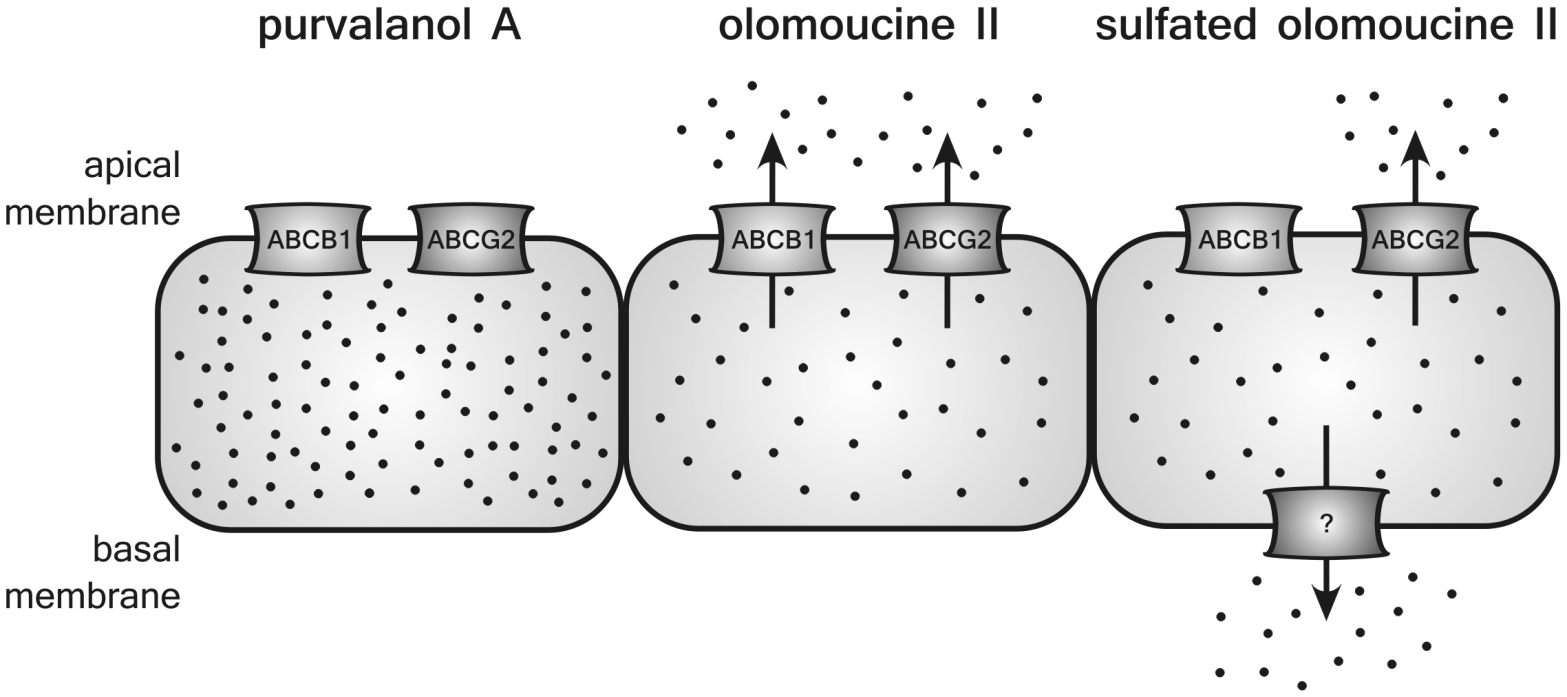
Schematic depiction of olomoucine II and purvalanol A transport in transduced MDCKII cells. Transport pathways for CDKi are indicated according to the results from MDCKII cellular monolayer transport assays. Transporter denoted with interrogation mark is unknown canine transporter.

In the present study, we investigated whether olomoucine II and purvalanol A are transported by ABCG2 and/or ABCB1. To date, only one paper has reported on substrate affinity of purine CDKi toward ABC transporters; using ATPase assay, vesicular transport, Hoechst 33342 and calcein assays, Rajnai et al. [31] demonstrated roscovitine to be a high affinity ABCB1 substrate and suggested that this interaction may be the reason for limited penetration of roscovitine across the blood-brain barrier. In addition, these authors concluded that roscovitine is not transported by ABCG2, multidrug resistance associated protein 1 (ABCC1) and multidrug resistance associated protein 2 (ABCC2). In our current work, using cellular monolayer transport assays with ABCG2 and ABCB1 transduced MDCKII cells, we demonstrate that olomoucine II is a dual substrate of ABCG2 and ABCB1 (Fig. 6). Based on our findings, it is feasible to presume considerable effect of both transporters on the pharmacokinetic behavior of olomoucine II, including absorption, distribution and excretion as well as limited uptake by tumors overexpressing ABCG2 and ABCB1. In addition, drug interactions with other substrates of these transporters must be considered in clinical use.

In contrast to olomoucine II, we show that purvalanol A is not transported by ABCG2 and ABCB1 *in vitro* (Fig. 6). In accordance with these results, we suggest that pharmacokinetic behavior and tumor treating abilities of purvalanol A will not be affected by ABCG2 and/or ABCB1. These findings may, at least partly, explain negligible resistance of ABCG2 overexpressing HeLa-6621 cells to purvalanol observed by Seamon et al. [32].

While investigating transport of olomoucine II across MDCKII monolayers, we detected time-dependent generation of a metabolite that we characterized as a sulfated conjugate of olomoucine II (Fig. 6). Enormous sulfatation capacity of MDCKII cells, significantly exceeding that of human liver, Chang liver and HepG2 cells, has previously been reported by Ng et al. [33]. Since transduced MDCKII cell lines are a well-established and widely used model in drug development for investigation of drug interactions with transport proteins [21], our findings are of great importance for other researchers performing transport or accumulation studies with MDCKII cells, especially if radiolabeled substrates are used. It is very likely that hydrophilic metabolites (sulfates) formed during the experiment will follow transport pathway(s) different from the parent compound and, eventually, may contaminate the pharmacokinetic analysis. Several endogenous canine transporters, such as Abcb1, Abcc1, Abcc2, and Abcc5, have been localized in the MDCKII cells [26], [27], of which multidrug resistance associated proteins can transport sulfated metabolites. We, therefore, assume that in our experiments, endogenous canine Abcc1, Abcc2 or Abcc5 transporters might efflux sulfated olomoucine II out of the MDCKII cells.

**Figure 7:**
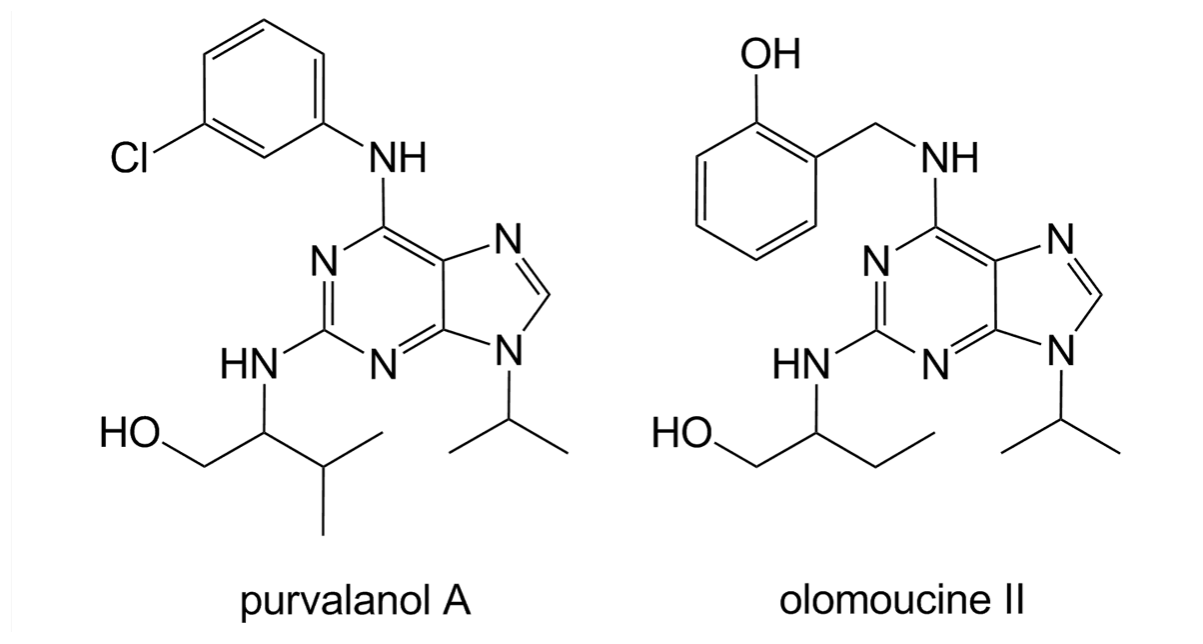
Chemical structures of olomoucine II and purvalanol A.

ABC transporters are well known for their ability to transport a wide variety of structurally unrelated molecules. Considering very similar structures of olomoucine II and purvalanol A (Fig. 7), it is surprising to see strikingly different interactions of both compounds with ABCB1 and ABCG2 proteins; only olomoucine II, but not purvalanol A, is transported by these transporters as observed in this study. However, our results correspond nicely with the studies by Ishikawa et al [34] or Nakagawa et al [35] who prepared several camptothecine analogues and tested them for their ability to circumvent the drug resistance mediated by ABCG2. The authors observed that analogues substituted with hydroxyl group were good ABCG2 substrates whereas replacement of the hydroxyl group with chlorine led to a remarkable reduction in affinity toward ABCG2. Correspondingly, olomoucine II (possessing hydroxyl group on the phenylamine substituent of purine heterocycle) was found to be a substrate of both ABC transporters in our study; on the other hand, purvalanol A (with –OH group replaced by chlorine) (Fig. 7) was not transported by any of the ABC transporters, suggesting that these two substituents play a key role in the recognition of the purine CDKi as ABC transporter substrates. Apart from *in vitro* experiments, we have confirmed identical behavior of both compounds on an organ level *in situ*; in perfused rat placenta, olomoucine II was actively pumped from fetus to mother by placental ABCB1/ABCG2 while purvalanol A showed no interactions with these transporters (data not shown) proposing our findings can be extrapolated beyond the *in vitro* experimental setup. It is thus apparent that structure similarity of particular CDKi cannot be used as a single reliable clue for the prognosis of interactions with ABC transporters.

## Conclusions

In conclusion, our data suggest that pharmacokinetic behavior of olomoucine II in the organism will be considerably affected by ABCG2 and ABCB1 transporters as well as by phase II biotransformation enzyme, sulfotransferase. Limited accumulation of olomoucine II in tumors overexpressing ABCG2 and ABCB1 can also be expected. At the same time, overlapping substrate specificity with other drugs may lead to drug-drug interactions on these transporters. In contrast, pharmacokinetic behavior of purvalanol A is not affected by either ABCG2 or ABCB1, theoretically favoring this drug in the treatment of tumors expressing efflux transporters. These facts should be taken into account when introducing these prospective compounds into the clinical area. In addition, care should be taken when performing pharmacokinetic studies in MDCKII cells, especially if radiolabeled substrates are used; sulfated conjugates formed within the cells may use other transport systems than the parent compound, which can eventually result in misleading interpretation of the pharmacokinetic analysis. With regard to chemical structures of olomoucine II and purvalanol A, our data emphasize that even drugs with remarkable structure similarity may show different pharmacokinetic behavior such as interactions with ABC transporters or biotransformation enzymes.
